# Medical Congresses

**Published:** 1903-08-22

**Authors:** 


### Medical Congresses.

It is not long since a medical congress was an event of the year, but such congresses are now so varied and numerous that they extend throughout the entire twelve months, and if anyone should desire to attend them all it would be necessary to traverse the whole world, and he need not then spend a single day without a séance. It is probable, however, that he would think his time might have been better spent, and at the end he would assuredly exclaim in the words of the Preacher, "Is there a new thing whereof men say, See, this is new? It hath been already in the ages which were before us. I have seen all the works that were done under the sun: and behold all is vanity and a striving after wind." The attendants at a congress may be divided roughly into the officers and those who picnic. The officers collect contributions which are either taken as read or are communicated to very small audiences: the picnickers are those who pay a subscription, obtain a distinguishing badge, and attend frankly for the purpose of enjoying themselves. To this end they throng the corridors and vie with each other in obtaining the greatest number of invitations for themselves and their wives. The gratuitous invitations are first snapped up, then the cheaper excursions, and, lastly, if the weather be promising, all the tickets are bought for everything that promises entertainment until the genuine worker, who often comes late because he has been busy in the particular section which interests him, finds to his disgust that there is nothing left for his gratification. The larger the congress, the greater is this grievance. At Madrid, the largest and the most unwieldy of all, because it was cosmopolitan and was held by a nation whose organising power, unfortunately, is not a strong point, an attempt was made to remedy this evil. Tickets for festivities where the numbers had to be limited were delivered to the presidents of each section with instructions that they should be issued to those who were present in the sections on a given day. The new method of distribution was good, but it failed precisely because it was new and because no notice of the method of issue was published until after the tickets had been actually distributed. Many fortunate individuals therefore found themselves in possession of coveted tickets which they had obtained by the accident of being in the section at the proper time.

But there are more serious evils attending congresses as they are at present held than the failure to obtain a ticket for an entertainment. The quantity of material offered is out of all proportion to the time at the disposal of the meeting. It follows therefore that many papers have to be taken as read which might profitably be discussed, whilst others, which are of merely academic interest, are read *in extenso* to an uninterested audience. This could be remedied to a large extent by enforcing much more strictly the rule that every contributor must furnish an abstract of his paper in sufficient time before the meeting to allow of its being printed and circulated. Many papers belie their title, and it is only by such means that the sectional officers are able to ascertain the true character of a contribution. They would then be able to group the papers, and so arrange them that those which promise to be productive of a good discussion should be brought forward early, ample notice being given of the time at which they will be read. This is the duty of the secretary; but the sectional president also has a proper sphere of action, and it is to see that each contributor adheres strictly to his allotted time. The difference between the success and failure of a section often depends upon this single point. A weak president allows a discursive speaker to continue *ad nauseam*, whilst a strong president keeps his members both to the point and the time. Again, at Madrid these methods were adopted, but they failed because the abstracts in many cases did not appear until after the paper had been communicated, and because, as the presidents were constantly changed in each section, there was no permanent authority to keep garrulous members in check.

Most of the medical congresses at the present time are too big. It is better to have a series of small congresses than one of gigantic size. A small congress is more personal; it brings together those who are interested in a given subject and allows them to become known to each other. It lessens the expense, also, because a monster congress means a raising of prices at lodgings and hotels. It enables the places of meeting to be more varied, for small towns can accommodate a small congress, whilst huge congresses have perforce to go to capital cities. But if a congress is to be small it should meet abroad as well as in England. Our insular limitations are still very great, and comparatively few medical practitioners in Great Britain know what is the routine practice in France or Germany. Travel is very cheap nowadays and it costs very little more to meet at Amiens or Bonn than in Liverpool or Glasgow. The question of language need not deter members. Many people can speak a little French or German, many German and French doctors speak English fluently, whilst, fortunately for our profession, there is no racial hatred amongst the better class of practitioners anywhere in Europe.

